# Principles of the prolactin/vasoinhibin axis

**DOI:** 10.1152/ajpregu.00256.2015

**Published:** 2015-08-26

**Authors:** Jakob Triebel, Thomas Bertsch, Cornelius Bollheimer, Daniel Rios-Barrera, Christy F. Pearce, Michael Hüfner, Gonzalo Martínez de la Escalera, Carmen Clapp

**Affiliations:** ^1^Institute for Clinical Chemistry, Laboratory Medicine and Transfusion Medicine, Paracelsus Medical University, Nuremberg, Germany;; ^2^Institute for Biomedicine of Aging, Friedrich-Alexander Universität Erlangen-Nürnberg, Nuremberg, Germany;; ^3^European Molecular Biology Laboratory, Developmental Biology Unit, Directors' Research, Heidelberg, Germany;; ^4^Southern Colorado Maternal Fetal Medicine, St. Francis Medical Campus, Centura Health, Colorado Springs, Colorado;; ^5^Endokrinologikum Göttingen, Göttingen, Germany; and; ^6^Instituto de Neurobiología, Universidad Nacional Autónoma de México (UNAM), Campus UNAM-Juriquilla, Querétaro, México

**Keywords:** prolactin, vasoinhibins, 16K prolactin, prolactin-fragments, prolactin/vasoinhibin axis

## Abstract

The hormonal family of vasoinhibins, which derive from the anterior pituitary hormone prolactin, are known for their inhibiting effects on blood vessel growth, vasopermeability, and vasodilation. As pleiotropic hormones, vasoinhibins act in multiple target organs and tissues. The generation, secretion, and regulation of vasoinhibins are embedded into the organizational principle of an axis, which integrates the hypothalamus, the pituitary, and the target tissue microenvironment. This axis is designated as the prolactin/vasoinhibin axis. Disturbances of the prolactin/vasoinhibin axis are associated with the pathogenesis of retinal and cardiac diseases and with diseases occurring during pregnancy. New phylogenetical, physiological, and clinical implications are discussed.

vasoinhibins are a novel family of hormones that are known for their antiangiogenic, antivasopermeability, and antivasodilation effects (16, 22–24, 31). Vasoinhibins derive from the pituitary hormone prolactin (PRL), as they are generated through the proteolytic cleavage of this hormone. PRL acts as a classical pituitary hormone, and, structurally, corresponds to a long-chain class-I helical cytokine. Like all other class-I helical cytokines, PRL folds into a bundle of four α-helices (57, 61, 94). Class-I helical cytokines signal via related receptors that share structural signatures (9, 95) and activate similar intracellular signaling pathways (93, 101). Despite their origin, vasoinhibins seem to have little in common with the typical characteristics of long chain class-I helical cytokines as they do not appear to activate similar intracellular signaling pathways and have different effects from their precursor counterpart (22, 23). Their set of vascular effects (inhibition of angiogenesis, vasodilation, and vasopermeability) is unique and entirely different from the effects of the precursor PRL molecule. It is this structural and functional distinctiveness that confers the identity to the vasoinhibin family.

Vasoinhibins, as defined by their characterization as a family, are not a single species, instead their molecular mass ranges between 11 and 18 kDa. This is due to the generation of vasoinhibins through proteolytic cleavage of PRL by several endogenous proteolytic enzymes, namely cathepsin D (8, 79), matrix metalloproteinases (69), and bone morphogenetic protein-1 (BMP-1) (45). Since these enzymes cleave full-length PRL at various sites near or within the long loop connecting the third and the fourth α-helix, proteolysis by these enzymes results in the synthesis of vasoinhibins of varying molecular masses. Vasoinhibins all share the NH_2_-terminal region of full-length PRL. The COOH-terminal fragment that arises from the proteolysis of PRL does not possess vasoinhibin-like activities (62).

The roots of discovery of vasoinhibins date back to the year 1980 when PRL fragments were first detected in extracts of the rodent pituitary cells (73). Soon thereafter, PRL fragments were found in the human pituitary gland and plasma (87). It was observed that PRL fragments are not only present in the pituitary gland, but are also generated in vitro by the rat prostate gland (27, 28), mammary gland, kidney, and liver (15, 108). At this time, their function was yet unknown. However, it was already assumed that their presence is not due to coincidental, unspecific breakdown of PRL, but instead that these PRL fragments have specific physiological significance (15). It was the discovery of the inhibiting effect that these PRL fragments exert on the proliferation of bovine capillary endothelial cells and human umbilical vein endothelial cells that first demonstrated their physiological relevance (19, 39). Continued work over the ensuing years provided further insights into the function of these PRL fragments, until, in 2006, they were characterized as a family and received the designation “vasoinhibins” inspired by one of their principal effects, the inhibition of angiogenesis (16, 17, 69). Today, vasoinhibins are known as a new family of hormones which, in addition to their physiological role in regulating vascular function and growth (23), also seem to be involved in the pathogenesis of diabetic complications (5, 44, 80, 98, 99), cancer (63, 74), and pregnancy-associated diseases (47, 51, 54, 64, 65, 70, 78).

## Generation, Secretion, and Regulation

Vasoinhibin generation in the pituitary gland occurs by proteolytic cleavage of their immediate precursor PRL by cathepsin D. Studies in rodents revealed that vasoinhibins are generated in the adenohypophysis, where, in the secretory granules of PRL, cathepsin D cleaves full-length PRL to yield vasoinhibins (34). Pituitary vasoinhibin generation is thus closely intertwined with PRL production. This is imperative, as vasoinhibins are not a product of a separate mRNA (73, 85), but are generated by a posttranslational modification, proteolytic cleavage, of PRL. However, the ratio of vasoinhibin generation to PRL synthesis is not fixed, instead it varies under physiological control. For example, the female, virgin rat features a pituitary vasoinhibin-to-PRL ratio of 0.22. During pregnancy, however, this ratio increases to 0.37 on *day* 9 and further to 0.77 on *day 12* (73). Consistent with these observations, plasma levels of vasoinhibins during the third trimester of human pregnancy are higher compared with nonpregnant states (87). The ratio of vasoinhibin to PRL synthesis is accessible to pharmacological manipulation, as demonstrated by the increase of the vasoinhibin-to-PRL ratio from 0.22 in the nontreated rat, to 0.99 after treatment with perphenazine, a dopamine D1 and D2 receptor antagonist that stimulates the production and release of PRL by the pituitary gland (73). The ratio is also increased by treatment with estrogen and reduced by thyrotropin releasing hormone (TRH) (34, 40, 73). Thus vasoinhibins possess the characteristics of effector hormones secreted from the anterior pituitary gland, similar to PRL, except that they feature posttranslational modification from the latter. These series of events, expression and posttranslational modification of PRL, and subsequent secretion by exocytosis resemble the generation of other hormones generated from a prohormone. Parallel examples are the generation of the adrenocorticotropic hormone (ACTH) and it's related peptide hormones generated in the corticotroph cells of the adenohypophysis by proteolytic cleavage of their precursor proopiomelanocortin (POMC) (105) and the thyroid hormone axis (TRH, TSH, T4/T3), where the bioactive T3 is processed from T4 by local deiodinases at the target tissue level.

It is also evident that vasoinhibin levels at the target tissue are regulated by the abundance of circulating PRL secreted by the pituitary gland. This is demonstrated by the observation that the induction of hyperprolactinaemia in rodents leads to vasoinhibin accumulation within the retina. Vice versa, pharmacological inhibition of pituitary PRL secretion with the dopamine agonist bromocriptine lowers the level of retinal vasoinhibins (5). Another situation in which vasoinhibin level at the target tissue depend on systemic PRL concentration is demonstrated in rodent studies investigating the role of vasoinhibins in peripartum cardiomyopathy (PPCM). Female mice receiving a chronic infusion of recombinant PRL had higher left ventricular myocardial vasoinhibin levels than controls (54).

The third tier at which vasoinhibin levels are regulated, next to intrapituitary regulation of the vasoinhibin-to-PRL ratio and total pituitary PRL secretion, is the expression and activity of the PRL-converting enzymes at the target tissue level. This is observed in mice with experimentally induced PPCM, where higher left ventricular vasoinhibin levels associate with increased protein concentration and activity of cathepsin D (54). This is also observed in the placenta of women with diabetes mellitus Type 1, in which, compared with controls, increased vasoinhibin generation associates with a higher expression of the PRL-cleaving enzyme BMP-1. Of note, placental mRNA PRL expression is also higher, suggesting that upregulation of local PRL synthesis can serve the purpose of providing sufficient amounts of substrate required for local vasoinhibin generation (78).

**Fig. 1.**
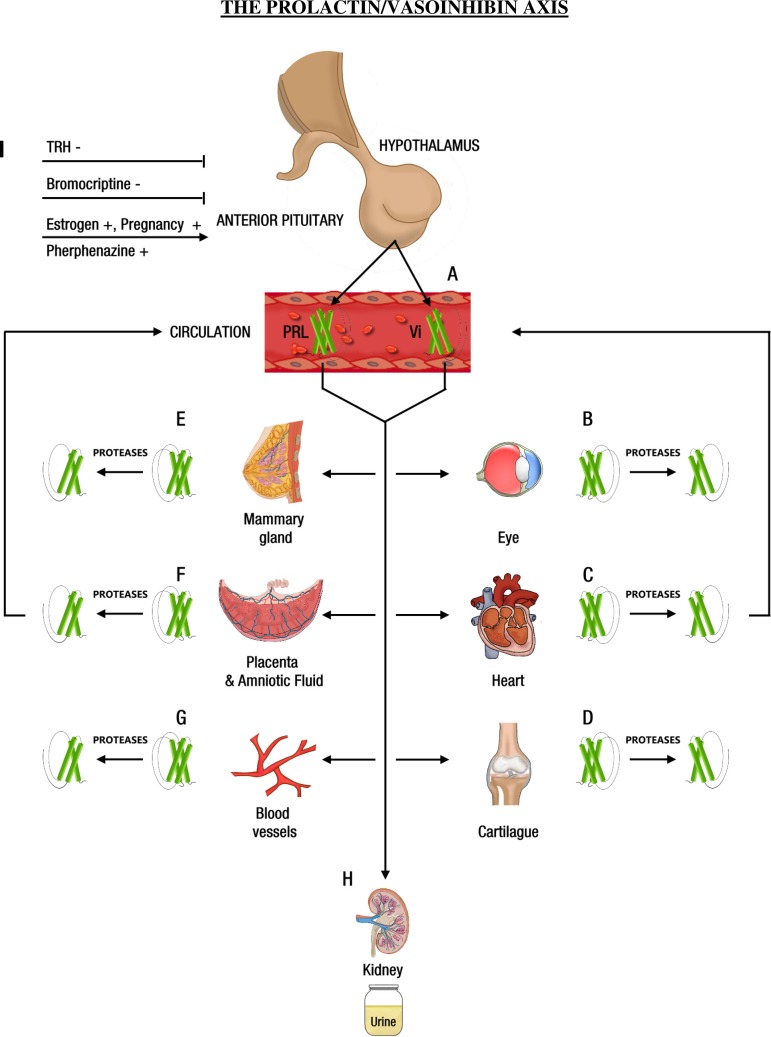
Central and peripheral regulation of the prolactin/vasoinhibin axis. *A*: anterior pituitary. Prolactin (PRL) and vasoinhibins (Vi) are secreted from the anterior pituitary gland. *B*: Eye. Hyperprolactinemia leads to vasoinhibin accumulation in the retina and inhibition of pituitary PRL secretion with the dopamine agonist bromocriptine lowers retinal vasoinhibins. A dysregulation of retinal/ocular vasoinhibins is linked to vasoproliferative retinopathies. *C*: Heart. Higher circulating PRL levels lead to higher left ventricular myocardial vasoinhibin levels. Local activity of cathepsin D regulates local vasoinhibin levels. An excessive myocardial vasoinhibin synthesis is linked to peripartum cardiomyopathy. Vasoinhibins generated in the heart can enter the circulation. *D*: Cartilage. Matrix metalloproteinases in cartilage generate vasoinhibins from circulating and cartilage-produced PRL. *E*: Mammary gland. Local activity of cathepsin D regulates local vasoinhibin levels. Vasoinhibins participate in mammary gland involution. *F:* Placenta and amniotic fluid. Local activity of bone morphogenetic protein-1 (BMP-1 and cathepsin D and upregulation of placental mRNA PRL expression regulate local vasoinhibin synthesis. An excessive, dysregulated placental vasoinhibin synthesis is linked to preeclampsia, fetal growth abnormalities, and maternal diabetes mellitus. Vasoinhibins generated in the placenta can enter the circulation. *G*: Endothelium. Endothelial cells express PRL mRNA and generate vasoinhibins. The role of circulating PRL and vasoinhibins on the levels of vasoinhibins at the endothelium is unclear. *H*: Kidney. Vasoinhibins appear in the urine of women with preeclampsia, pointing toward an altered renal elimination of vasoinhibins under pathophysiological conditions. *I*: Central regulation. Thyrotropin releasing hormone (TRH) and bromocriptine inhibit the synthesis of vasoinhibins in the anterior pituitary gland. Estrogen, the state of pregnancy and the antipsychotic drug perphenazine stimulate the synthesis of vasoinhibins in the anterior pituitary.

Three principles can be derived from the above observations. First, vasoinhibins are generated in the anterior pituitary gland from which they are secreted as effector hormones. Second, the central, anterior pituitary generation of vasoinhibins is under physiological control over the total anterior pituitary PRL production and activity of the PRL converting proteases. Third, the regulation of vasoinhibin concentration at the target tissue level includes the utilization of circulating and locally produced PRL and the level of activity of local PRL cleaving enzymes. Thus it appears that generation, secretion, and regulation of vasoinhibin action demonstrate the organizational principle of an axis that integrates the hypothalamus, the pituitary, and the target tissue level (Fig. 1). Indeed, this corresponds with the classical three tiers of control that subserve the regulation of anterior pituitary hormone secretion (82). Furthermore, the generation of vasoinhibins at the target tissue level represents a novel example of paracrinology, an exciting concept in endocrinology by which hormone action is being regulated at the target tissue microenvironment (56).

## Molecular Species, Distribution, and Target Tissues

**Fig. 2.**
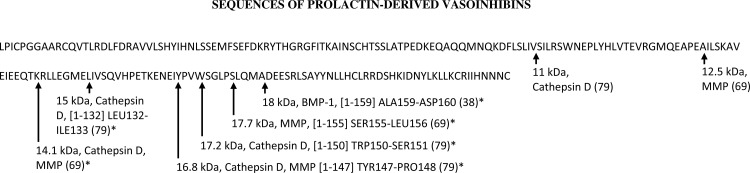
The protein sequences of full-length PRL and of PRL-derived vasoinhibins are presented, including information on their molecular mass and enzyme involved in the vasoinhibin generation and cleavage sites (indicated with arrows). The number in brackets indicate the residues comprising the respective vasoinhibin molecule. Where available, the cleavage site is also indicated. *Vasoinhibins that have been tested for anti-angiogenic activity.

Cleavage of human PRL by cathepsin D results in the generation of vasoinhibins comprising residues 1-80-85 (11 kDa),1–150 (17.2 kDa), 1–147 (16.8 kDa), and 1–132 (15 kDa) (79). Cleavage of PRL by matrix metalloproteinases (MMP) results in vasoinhibins comprising residues 1–150 (17.7 kDa), 1–132 (16.8 kDa), 1–124 (14.1 kDa), and 1–111 (12.5 kDa) (69). MMP cleave PRL to generate vasoinhibins with the following relative potency: MMP-8 > MMP-13 > MMP-3 > MMP-1 > MMP-2 > MMP-9 (69). Proteolysis of PRL by BMP-1 generates a single vasoinhibin species comprising residue 1–152 (17 kDa) (45) (Fig. 2). Oxidative stress can increase the activity of cathepsin D to cleave PRL (54), whereas hypoxia decreases cathepsin D-induced vasoinhibin generation (33). A higher expression of cathepsin D and a parallel increase in vasoinhibin generation is also observed during mammary gland involution (58). The activity of MMP from chondrocytes to generate vasoinhibins seems to be higher in patients with arthritis (69), pointing toward the possibility of an induction of vasoinhibin generation by inflammatory factors. Factors associated with the activity of BMP-1 in the generation of vasoinhibins are not known.

Vasoinhibin species are detected in the rat (34, 73), mouse (86), and human (79, 87) pituitary gland, the human vascular endothelium (30), the human and rat placenta (78), the bovine corpus luteum (38), and the following rodent tissues: hypothalamus (110), neurohypophysis (25), cartilage (69), and retina (4, 5). However, the placental localization of vasoinhibins may be questioned since placental samples can be contaminated with decidua, and both human and rodent decidua produce PRL (52, 76). Of note, in vitro studies demonstrate that these and the following tissues generate vasoinhibins when exposed to full-length PRL: heart (54), ventral prostate, spleen, lung, kidney and mammary gland (8, 15, 27, 28, 58, 108) in rodents, and the mammary gland (7) and placenta (47) in humans. Vasoinhibin species are also detected in human and rat plasma/serum (37, 47, 87, 98), in human amniotic fluid (43, 47), subretinal fluid (37), and urine (47, 65). A summary of the distribution and functions of vasoinhibins in the human and the rodent organism is presented in Table 1.

**Table 1.** Distribution and functions of endogenous vasoinhibins in humans, rodents, and other organisms

	Endogenous Vasoinhibins		Vasoinhibin Generation		Vasoinhibin Mass (kDa)/Protease			
Anatomical Location	Human	Rodent	Human	Rodent/other	Human	Rodent	Established or *Assumed* Functions	Ref. No.
*Central Nervous System*								
Pituitary gland	yes	yes	—	yes	16	16/CD	Generation, secretion, ↓ proliferation, ↑ apoptosis	34, 73, 79, 86, 87
Neurohypophysis	—	yes	—	yes	—	14	*VEGF-antagonism*	25
Hypothalamus	—	yes	—	yes	—	14	*VEGF-antagonism*	(25, 71, 110)
							↑ Anxiety, ↑ depression	
						17	↑ Vasopressin release	
Retina	—	yes	—	yes	—	16	↓ Permeability, ↓ vasodilation	(4, 5)
Fibrovascular membranes	yes	—	—	—	16	—	↑ Vascular regression	(37)
*Body Fluids*								
Plasma/serum	yes	yes	—	—	18; 16; 14	14	Transport, ↓ *vasopermeability*, ↓ *vasodilation*	(31, 37, 47, 87, 96, 98)
Amniotic fluid	yes	—	yes	—	14/CD	—	VEGF-antagonism	(43, 47)
Subretinal fluid	yes	—	yes	—	16/NP	—	↑Vascular regression	(37)
Urine	yes	—	—	—	18; 16; 14	—	*Elimination*	(47)
*Organs/Tissues*								
Placenta	yes	yes	yes	—	17	16	*Anti-angiogenesis*	(47, 70, 78)
					15	14		
Mammary gland	—	yes	—	yes	—	16, 14/CD	↑ Involution	(15, 58, 108)
Corpus luteum	—	yes	—	yes/bovine	—	16/CD	*↑ Angioregression*	(38)
						14/CD		
Endothelial cells	yes	—	yes	yes/bovine	16, 14	—	↓ Proliferation, ↓ migration	(18, 30)
Fibroblasts	—	yes	—	yes	—	17/BMP-1	Antiangiogenesis	(32, 45, 69)
						16/CD		
Heart	—	—	—	yes	—	16/CD	↓ Cardiomyocyte metabolism, anti-angiogenesis	(54)
Liver	—	—	—	yes	—	16	Unknown	(15)
Cartilage/chondrocytes	—	—	yes	yes	17,16,14/MMP	17,16,14/MMP	*Antiangiogenesis*	(69)
						14/CD		
Prostate	—	—	—	yes	—	16/CD	Unknown	(27, 28)
Testes/sperm	—	yes	—	—	—	16,17,18	Unknown	(59)

The anatomical locations with corresponding evidence in regard to the occurrence of endogenous vasoinhibins, the ability to generate vasoinhibins from prolactin (PRL), the vasoinhibin molecular species, and the protease involved are presented. Established functions relates to in vitro and/or in vivo evidence in the cited literature, whereas assumed function (in italic) relates to putative functions not supported by in vitro or in vivo data. CD. cathepsin D, MMP. matrix metalloproteinases; NP, neutral proteases. –Not reported.

The presence of vasoinhibins in the pituitary gland and plasma reflect their hormonal nature, that is, their generation and secretion by an endocrine gland into the circulation. However, vasoinhibins can also act locally at their production site, as demonstrated in rodent studies in which antiproliferative and proapoptotic actions of vasoinhibins were observed in the anterior pituitary gland (40). In case of the hypothalamic paraventricular nuclei and the supraoptic nucleus, it appears that vasoinhibins function as stimulators of vasopressin release (71) and as antagonists of vascular endothelial growth factor (VEGF), whose expression is particularly high at these sites (25, 60). An antagonism to the effects of VEGF may also operate in cartilage, an avascular tissue (69), and is observed in the rat retina, in which vasoinhibins reduce VEGF- and diabetes-induced retinal vasopermeability (5, 44). Indeed, because of the close anatomical vicinity of vascularized tissues and avascular compartments, the eye, in which vasoinhibins control ocular angiogenesis, vasodilation, and vascular permeability, is one of the best-characterized target organs for vasoinhibins and represents a key illustrative example of the actions of vasoinhibins (4, 21, 22, 44, 77, 99). However, as these functions of vasoinhibins and their clinical implications in retinal diseases have recently been reviewed (21, 22, 99), they will not be subject of this review. Rats receiving the intracerebroventricular administration of vasoinhibins show anxious and depressive behaviors (110), suggesting that vasoinhibins may be local regulators of neuronal function.

Whether the effects of vasoinhibins in the above-mentioned target tissues are due to their action on the vessel system in these tissues or on a tissue-specific cell-type is not always clear. The vasoinhibin effect in the heart, however, in which vasoinhibins utilize the cardiomyocyte to exert their effect on the heart's vasculature, points to the possibility of a complex interplay between both, vascular and nonvascular cells. Furthermore, the proportion as to which the total amount of vasoinhibins present in a target tissue is of systemic or local origin is unknown.

## Recognition, Relay, and Elimination

PRL signals through the binding-induced dimerization of single transmembrane receptors that activate various kinases including its canonical Janus kinase 2 (JAK2)-signal transducer and activator of transcription (STAT) pathway, the phosphatidylinositol 3-kinase (PI3K)/AKT pathway, and the mitogen-activated protein kinase (MAPK) pathway (11). Of note, PRL is produced by endothelial cells (24, 109) and uses the JAK2-STAT5 pathway to promote their migration and tube formation (83, 109) and to stimulate the expression of proangiogenic factors (FGF-2 and VEGF) by various nonendothelial cells (40, 41), suggesting that PRL acts not only as a systemic, but also as an autocrine/paracrine positive regulator of angiogenesis. On the other hand, vasoinhibins inhibit angiogenesis, vasodilation, and vasopermeability by mechanisms that include blocking the activation of the Ras-Raf-MAPK pathway, the Ras-Tiam1-Rac1-Pak1 pathway, and the Ca^2+^/calmodulin-activation of endothelial nitric oxide synthase (eNOS) (46). They also promote protein phosphatase 2A-induced dephosphorylation/inactivation of eNOS (44), the activation of proapoptotic proteins of the Bcl-2 family, and the nuclear factor-κB (NFκB)-mediated activation of caspases. These and other intracellular signaling pathways of vasoinhibins have been reviewed recently (22, 23). The exact nature of the mechanism by which vasoinhibins interact with their target cells to activate these signaling pathways is unclear. However, the discovery of high affinity vasoinhibin binding sites on bovine brain capillary endothelial cells was reported in 1992 (26). These vasoinhibin-binding sites have a high binding affinity, are saturable, and specific. Cross-linking experiments identified 52-kDa and 32-kDa proteins as the major vasoinhibin binding species. However, until today, the exact identity of these vasoinhibin putative receptors has not been revealed.

A target (66) and frequent binding partner of vasoinhibins is plasminogen activator inhibitor-1 (PAI-1). PAI-1 is a serine protease inhibitor that by inhibiting tissue-type plasminogen activator (t-PA) and urokinase-type plasminogen activator (u-PA) blocks the generation of plasmin from plasminogen and suppresses clot dissolution (36). PAI-1 forms a complex with uPA and uPAR on endothelial cell membranes (12). Of note, vasoinhibins upregulate PAI-1 expression in endothelial cells (66) and were recently reported to form a complex with endogenous PAI-1 in the culture medium of these cells, as well as in human and mouse plasma (6). Vasoinhibins also colocalized with the PAI-1-uPA-uPAR complex on the endothelial cell surface and formation of such multiprotein complex was required for the antiangiogenic activity of vasoinhibins. This was concluded from experiments demonstrating that silencing or pharmacological blockage of PAI-1 and uPAR signaling abrogated the antiangiogenic effect of the vasoinhibins in HUVEC (6). Of note, the apparent molecular weight of the binding proteins (52 kDa and 32 kDa) of the above-mentioned high affinity vasoinhibin binding site (26) is close to the molecular masses of PAI-1 (43 kDa), uPA (51 kDa), and uPA receptor (55 kDa), and their identity could indeed be the PAI-1-uPA-uPAR complex. However, the specific binding affinity of vasoinhibins to these endothelial membrane species was much higher (*K*_d_ = 10 nM) than to PAI-1 (1 μM) or uPA-PAI-1 (0.5 μM). While the difference in affinity may suggest a higher *K*_d_ for the natural multicomponent PAI-1-uPA-uPA receptor complex, the contribution of other vasoinhibin binding sites, for example a new cell surface receptor, cannot be disregarded and continues to be an unresolved question.

A role further downstream in the vasoinhibin-signaling cascade, downstream from a potential cell surface receptor or signaling via PAI-1-uPA-uPAR, is described for the vasoinhibin-miR146a (microRNA-146a) circuit. It appears from the studies in PPCM models, that vasoinhibins mediate part of their antiangiogenic effects via induction of miR146a in endothelial cells. If exposed to vasoinhibins, HUVEC proliferation is reduced via upregulation of miR146a and HUVEC release miR146a-loaded exosomes that can be absorbed by neonatal rat cardiomyocytes (51). In these cardiomyocytes, miR146a has a detrimental effect and reduces cardiomyocyte metabolic activity, an effect that presumably contributes to the pathogenesis of PPCM (51). Both anti-miR146a transfection and silencing or pharmacological blockage of PAI-1 and uPAR signaling result in an abrogation of the vasoinhibin antiangiogenic effect. This implies that one of these signals has to be regulated by the other, and further studies are required to characterize this regulatory loop. The other two prominent vascular effects of vasoinhibins besides inhibition of angiogenesis, inhibition of vasodilation, and vasopermeability are effects mediated by vasoinhibin-induced blockage of eNOS activity (44, 46). It remains to be shown whether vasoinhibins utilize the miR146a-circuit and/or interaction with PAI-1 to downregulate eNOS activation.

No data exist about the half-life of vasoinhibins in the circulation. The extent as to which the liver and the kidney contribute to the clearance of vasoinhibins is also unknown. However, vasoinhibins were detected in the urine of women with preeclampsia (47, 65), pointing toward the possibility of an altered renal elimination of vasoinhibins under pathophysiological conditions.

## New Physiological and Clinical Implications

New physiological and clinical implications of the prolactin/vasoinhibin axis arise from the governing principle of the growth-restricting effects of vasoinhibins. Vasoinhibins mediate their growth-restricting effects via the inhibition of angiogenesis, which reduces tissue growth due to the lowering of oxygen and nutrient availability. Physiological tissue growth and angiogenesis is fundamental in pregnancy, where angiogenesis is a prerequisite for growth of the placenta and therefore fetal growth.

During pregnancy, PRL level progressively rise to ∼20–40 ng/ml at the end of the first trimester, 50–150 ng/ml by the end of the second trimester, and 100–400 ng/ml at term (100). This rise in circulating PRL level is accompanied by a rise in circulating vasoinhibin level (87), perhaps due to the increased PRL availability for pituitary vasoinhibin generation and release (73). Another source of PRL during pregnancy is the decidual tissue, especially at the maternal-fetal interface (76). While the known physiological purpose of the increase in PRL levels includes the preparation of the breast for lactation, the physiological effect of the increase in vasoinhibin level is less clear, particularly since an elevation of vasoinhibin levels is associated with diseased pregnancy states and adverse pregnancy outcomes.

Vasoinhibins are generated in the human placenta with higher rates observed in placentas from women with preeclampsia (70) and Type 1 diabetes mellitus (78). Furthermore, vasoinhibins are elevated in serum, urine, and amniotic fluid of women with preeclampsia compared with normotensive controls, and their occurrence in urine increases with the severity of the disease and the occurrence of adverse outcomes (47, 64, 65). In fact, the presence of vasoinhibins in urine predicts adverse maternal and perinatal outcomes. The odds ratio in women with preeclampsia and urinary vasoinhibins for combined adverse maternal outcomes (pulmonary edema, acute renal failure, placental abruption, hepatic hematoma or rupture, intubation required, and use of inotropics) is 44.9 (95% CI 5.1–392.3), for stillbirths or neonatal deaths is 1.3 (95% CI 0.4–3.6), and for small for gestational age infants is 1.9 (95% CI 1.1–3.1) (64). The antiangiogenic effects of vasoinhibins and the paramount importance of an adequate vascularization of the placenta imply a causal link between vasoinhibins and these outcomes. Furthermore, human and rat placental tissue featuring higher vasoinhibin synthesis in the presence of maternal diabetes demonstrates hypovascularization with reduced vascular surfaces and capillary density (78). Also, the level of vasoinhibins in amniotic fluid from patients with preeclampsia is inversely correlated with birth weight, that is, higher vasoinhibin level in the amniotic fluid associates with lower birth weight (47), and vasoinhibins antagonize the proangiogenic actions of VEGF in amniotic fluid from patients with preeclampsia (47). These outcomes as well as the pregnancy-associated diseases of preeclampsia and PPCM share a common milieu of increased inflammation, a factor in the activation of the proteolytic enzyme cathepsin D. Therefore, it is conceivable that a higher placental synthesis of vasoinhibins, combined with their increased pituitary release into the circulation during pregnancy, contributes to abnormal placental vascularization and thus to intrauterine growth restriction and small for gestational age infants. Indeed, the regulation of factors stimulating and inhibiting growth between the maternal organism and the fetoplacental unit is usually resolved at the level of the maternal reproductive tract. However, in the fetoplacental unit, a pathophysiological increased synthesis of vasoinhibins may disrupt the balance of growth factors and their inhibitors that contribute to adverse maternal and perinatal outcomes. This disruption is also speculated to occur at the level of organ dysfunction seen in association with increased vasoinhibins, such as the cardiac myocyte [PPCM (54, 55)], the endothelium [preeclampsia (47, 64)], the kidney [preeclampsia (47, 64)], and as above, the placenta (preeclampsia, fetal growth abnormalities). Clinical studies in women with PPCM using bromocriptine to suppress PRL secretion and cardiac vasoinhibin synthesis point toward beneficial effects, and a randomized, multicenter clinical trial is currently under way (49, 50, 53, 88).

## Unresolved Questions

**Fig. 3.**
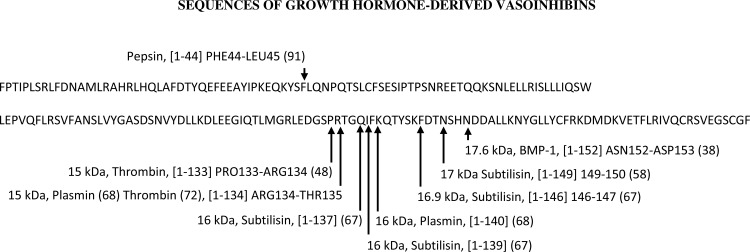
The protein sequences of full-length growth hormone (GH) and of GH-derived vasoinhibins are presented, including information on their molecular mass and enzyme involved in the vasoinhibin generation and cleavage sites (indicated with arrows). The number in brackets indicate the residues comprising the respective vasoinhibin molecule. None of these GH-related vasoinhibins have been tested for anti-angiogenic activity.

**Fig. 4.**
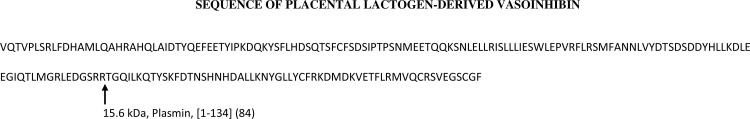
The protein sequences of full-length placental lactogen (PL) and of PL-derived vasoinhibins are presented, including information on their molecular mass and enzyme involved in the vasoinhibin generation and cleavage site (indicated with arrow). The number in brackets indicate the residues comprising the vasoinhibin-molecule. The PL-derived vasoinhibin has not been tested for anti-angiogenic activity.

Prolactin and growth hormone (GH) are phylogenetically related, they evolved from a common ancestral gene (75), and share a long-chain class-I helical cytokine structure comprising four α-helices. Placental lactogen (PL), the third member of the GH/PRL/PL-family, and another class-I helical cytokine arose independently by duplication of the PRL gene in rodents and ruminants and by duplication of the GH gene in primates (42). GH and PL likely contribute to the generation of endogenous vasoinhibins (Figs. 3 and 4); however, the generation and effects of GH- and PL-derived vasoinhibins have received little attention. Recombinant vasoinhibins derived from GH and PL displayed antiangiogenic effects in vitro (92). However, no information about their endogenous generation and clinical relevance is available. Importantly, GH-derived, vasoinhibin-like fragments were not detected in the rat pituitary, suggesting that any possible, endogenously GH-derived vasoinhibins may not be generated in the pituitary gland (73). Thus the contribution of GH and PL cleavage resulting in the synthesis of vasoinhibins to the function or regulation of the prolactin/vasoinhibin axis is unclear. The observation that GH is not converted to vasoinhibins in the rat pituitary, suggests that the possible contribution of GH to the total endogenous vasoinhibin level is restricted to the peripheral tissue level. Placental lactogen is increased in pregnancy and is produced by the human placenta. It is not known whether PL is converted to vasoinhibins at the level of the placenta as well.

It is unclear if the differences in molecular mass impact the structure and function of vasoinhibins. Also, it remains to be shown whether and to what extent each of the various proteases contributes to the physiological, endogenous level of vasoinhibins, and how the synthesis of different vasoinhibins is modified in disease conditions. Accordingly, the total composition and full identity of endogenous vasoinhibins in the circulation and at the target-tissue levels, including their interplay, has yet to be determined. The impact of glycosylation of PRL on vasoinhibin physiology is unknown. It is possible, however, that glycosylation alters the proteolytic cleavage of PRL and may thus impact vasoinhibin generation, as well as the action and clearance of these peptides (10). These unresolved questions have delayed the development of a uniform classification/nomenclature for vasoinhibins (97).

Another major unresolved question is the normal concentration of endogenous vasoinhibins in the human circulation. This is owing to the lack of a quantitative assay, with which circulating vasoinhibin levels and reference ranges could swiftly be established. The only method for the evaluation of endogenous vasoinhibins, which is considered relatively reliable is immunoprecipitation and Western blotting. However, this method is only semiquantitative and subject to significant interassay variability. Mass spectrometric techniques were successfully adapted to determine vasoinhibins in sera (106); however, these techniques are experimental and not commercially available. The urgent need for a quantitative assay for vasoinhibins is further underscored by the clinical implications of vasoinhibins in diabetic complications, cancer, and pregnancy-associated diseases, in which the quantitation of circulating vasoinhibins are of relevance for diagnosis, treatment, and risk stratification.

## Phylogenetic Context

Prolactin is a pleiotropic hormone whose functions are classified into seven categories: water and electrolyte balance, growth and development, endocrinology and metabolism, brain and behavior, reproduction, immunoregulation and protection, and actions associated with pathological disease states (13). As vasoinhibins are structurally and functionally distinct from PRL, their function is not considered in the classification of PRL biological effects. However, since PRL constitutes the precursor of vasoinhibins, and their regulation and function is embedded into the prolactin/vasoinhibin-axis, the effects of vasoinhibins cannot be comprehended without the consideration of PRL biology. Remarkably, vasoinhibins feature actions in the same categories. It derives from this consideration, that the evolutionary forces that governed PRL phylogenesis may have also determined the emergence of vasoinhibins.

Prolactin, GH, and PL have evolved from a common ancestral gene. On the basis of sequence comparisons of tetrapod hormones, the divergence is located approximately 392 million years ago (29). Two models for the function of the ancestral gene that gave rise to the GH/PRL family have been proposed. The first model proposes that the function of the ancestral gene was to regulate somatic growth (90), while the opposing model proposes that the gene was involved in osmoregulation because this function is common to fish PRL and GH (14). The third member of the somatotropin/prolactin family in fish, besides PRL and GH, is somatolactin (3, 81). The significance of somatolactin as an ancestor of the GH/PRL family in higher vertebrates in the context of the vasoinhibin physiology is not known. However, sequence analyses of somatolactin and human PRL demonstrates a low consensus, and none of the known cleavage sites of human PRL that are required for the generation of vasoinhibins are present in somatolactin.

Since the emergence of the PRL gene from its ancestor, periods of rapid change within the coding sequence of mature PRL lead to substantial sequence differences between human PRL and that of nonprimate mammals and other species (104). There is no evidence for this divergence creating an overall loss of function of PRL in higher primates, making an increased acceptance of mutations associated with a loss of function unlikely (104). Instead, Wallis et al. argue that it is more likely that the periods of rapid change corresponded to periods in which physiological functions of PRL varied and slowing the rate of evolution accompanied a stabilization of the biological function. Furthermore, Wallis et al. (104) propose that the nonrandom distribution of substitutions, coupled with no major loss of function supports positive selection as the most probable explanation for the episodes of rapid evolution. The gain of function of PRL that must have occurred during this process has not been elucidated. However, such gain of function of PRL could have corresponded with the emergence of the ability of PRL to function as a precursor for vasoinhibins. This view is supported when comparing the cleavage sites present in human PRL across taxa. An orthologue comparison with a protein sequence alignment of representative species of several major taxons is presented in Table 2. Indeed, while five cleavage sites are present in the human and the gorilla PRL, four cleavage sites are present in the macaque, three in the pig and the oppossum, two in the duck and in the *Xenopus tropicalis*, and none in the zebrafish and the spotted gar (Table 2). It appears, thus, that vasoinhibins emerged in tetrapods and that throughout speciation, there was an increase in cleavage sites with the highest number of cleavage sites present in primate species. Along this line, the ability of vasoinhibins to balance blood vessel growth, function, and involution could have contributed to PRL effects throughout vertebrate phylogeny and to its value as a biologically conserved factor (1, 20). A limiting factor is that the selected species may represent exceptions rather than representatives of the rule. A full comparative genomics approach will help to strengthen this analysis.

**Table 2.** Prolactin cleavage sites required for vasoinhibin generation in different species

		**Protein-Alignment Within Cleavage Site Regions**					
		Cathepsin D			MMP	BMP-1	
**Taxon**	**Species**	15 kDa Vi	16.8 kDa Vi	17.2 kDa Vi	17.7 kDa Vi	18 kDa Vi	**Cleavage Sites**
Teleost fishes/*Otophysa*	Zebrafish	…GLEHVVHK…	…LSTLPFNG…	…STLPFNGN…	…GNNLGQDK…	…LGQDKTSR…	0
Ray-finned fishes/*Neopterygii*	Spotted gar	…GVEKVAEK…	…SSA- - - -…	…SA- - - - DA…	…-DALLPSA…	…PSSASNDA…	0
Lobe-finned fishes/*Sarcopterygii*	Coelacanth	…GMECIVGQ…	...SELQAPWP…	…QAPWPG-P…	…G-PLLLLD…	…LLLDGEDQ…	0
Tetrapods/*Tetrapoda*	*X. tropicalis*	…GMEKIVGR…	…NDVNSLWS…	…**NSLW-SGPP**…	…GPMAAQSA…	**…AQSA-DENS…**	2
Reptiles and birds/*Sauria*	Duck	…GMEKIVGR…	…NEIYSQWE…	…ISQWEGLP…	**…GLPS-LQLA…**	**…LQLA-DEDS…**	2
Marsupials/*Marsupialia*	Opossum	…GMEKIVGQ…	…NEVYSVWS…	**…ISWV-SGLP…**	**…GLPS-LQMA…**	**…LQMA-DEDT…**	3
Laurasiatherian Mammals/*Laurasiatheria*	Pig	…GMEKIVGQ…	…NEVYSVWS…	**…YSVW-SGLP…**	**…GLPS-LQMA…**	**…LQMA-DEDT…**	3
Old World monkeys/*Cercopithecinae*	Macaque	**…GMEL-IVSQ…**	**…NEIY-PVWT…**	…YPVWTGLP…	**…GLPS-LQMA…**	**…LQMA-DEES…**	4
Great apes/*Hominidae*	Gorilla	**…GMEL-IVSQ…**	**…NEIY-PVWS…**	**…YPVW-SGLP…**	**…GLSP-LQMA…**	**…LQMA-DEES…**	5
Great apes/*Hominidae*	Human	**…GMEL-IVSQ…**	**…NEIY-PVWS…**	**…YPVW-SGLP…**	**…GLSP-LQMA…**	**…LQMA-DEES…**	5

A PRL orthologue comparison using a human PRL protein sequence alignment of representative species of major taxons. The protein sequences were retreived from the ENSEMBL genome browser (http://www.ensembl.org/index.html), release 79, March 2015 (35, 41). The protein sequence alignment shows the regions at which proteolytic cleavage of full-length PRL occurs, resulting in the generation of vasoinhibins (Vi). Regions with cleavage sites are marked in boldface and the cleavage site (marked with -) and the neighboring four NH_2_-terminal and COOH-terminal amino acids are shown. No cleavage sites were found in representative species of teleost, Ray-finned, and Lobe-finned fishes. Two cleavage sites are found in the PRL sequence of the *X. tropicalis*, a different set of two cleavage sites in the duck, and three, four, and five cleavage sites are present in representative species of marsupials, Laurasiatherian mammals, Old World monkeys, and Great apes, respectively.

An additional level of complexity, beyond the scope of this review, is the contribution of other members of the PRL family in rodents, such as proliferins and proliferin-related proteins known to have effects on blood vessels (31, 89). This also applies for species-specific GH or PRL family gene expansions in different species including primates, rodents and ruminants (2, 102, 103, 107).

## Perspectives and Significance

The process of vasoinhibin generation and secretion from the anterior pituitary gland appears to be under physiological control through mechanisms affecting the biosynthesis of PRL and the expression/activity of PRL cleaving proteases. Also, the circulating PRL levels and the local synthesis of PRL affect the peripheral generation of vasoinhibins at the target tissue level. Therefore, the generation, secretion, and regulation of vasoinhibin action are embedded into the organizational principle of an axis, which integrates the hypothalamus, the pituitary, and the target tissue microenvironment. On the basis of the established nomenclature for other endocrine axes, this axis is designated as the prolactin/vasoinhibin axis. The significance of the prolactin/vasoinhibin axis in pathophysiological states is surfacing to provide a better understanding of the role of PRL in clinically apparent disease conditions. Among these, diseases associated with the state of pregnancy are most prominent as profound changes in PRL metabolism during pregnancy and postpartum seem to render the prolactin/vasoinhibin axis particularly susceptible for disturbances that would cause major changes in the production/action of vasoinhibins. Recent studies concerned with the search for vasoinhibin signaling mechanisms, and particularly with the cell surface receptor involved in their actions, are providing valuable insights into the complexity of vasoinhibin actions within the vascular and nonvascular microenvironment.

This review brings into focus major research challenges needed to advance the field in the near and mediate future. A systematic approach is required to help identify the physiologically relevant vasoinhibin molecules and the cleaving proteases that generate them. This approach should include the characterization through uniform conditions of their antiangiogenic, antivasopermeability, antivasodilatatory effects, and signaling mechanisms. This knowledge will help develop a proper nomenclature for the definition of the various endogenous vasoinhibin isoforms and the development of a quantitative assay capable of differentiating them and their precursor proteins (PRL, GH, PL) in body fluids. The latter is a major demand for studying the involvement of vasoinhibins in pregnancy-associated diseases, diabetic complications, and cancer and for their implications in risk stratification, diagnosis, and treatment. Naturally, resolving the solution structure of vasoinhibins by NMR spectroscopy, possibly by analyzing recombinant human vasoinhibins, would enhance our understanding of the structure-function relationships of the vasoinhibin family and produce valuable insights into their mechanism of action. Upon successful resolution of these issues, the knowledge of the role of vasoinhibins in health and disease will be greatly enhanced and the development of a sustainable classification of vasoinhibins that provides orientation in future biomedical research, will commence.

## GRANTS

This study was supported by the National Council of Science and Technology of Mexico (CONACYT, Grant SALUD-2012-1-179506 to C. Clapp).

## DISCLOSURES

No conflicts of interest, financial or otherwise, are declared by the author(s).

## AUTHOR CONTRIBUTIONS

Author contributions: J.T. and C.C. conception and design of research; J.T., D.R.-B., C.F.P., and C.C. analyzed data; J.T. and C.C. interpreted results of experiments; J.T., T.B., D.R.-B., G.M.d.l.E., and C.C. prepared figures; J.T. drafted manuscript; J.T., T.B., D.R.-B., C.F.P., M.H., G.M.d.l.E., and C.C. edited and revised manuscript; J.T., T.B., C.B., D.R.-B., C.F.P., M.H., G.M.d.l.E., and C.C. approved final version of manuscript.
